# Safety of intraarticular corticosteroid injection preceding hip and knee arthroplasty: a systematic review and meta-analysis amid resolving COVID-19 arthroplasty restrictions

**DOI:** 10.1093/jhps/hnab064

**Published:** 2021-08-24

**Authors:** Tim Cheok, Matthew Jennings, Alessandro Aprato, Narlaka Jayasekera, Ruurd L Jaarsma

**Affiliations:** Department of Trauma and Orthopaedics, Alice Springs Hospital, 6, Gap Road, Northern Territory 0870, Australia; Department of Trauma and Orthopaedics, Alice Springs Hospital, 6, Gap Road, Northern Territory 0870, Australia; Traumatologic Hospital, University of Turin, via Gianfranco Zuretti, 29, Turin 10126, Italy; Department of Trauma and Orthopaedics, Alice Springs Hospital, 6, Gap Road, Northern Territory 0870, Australia; Flinders Medical Centre, Flinders Drive, Bedford Park, South Australia 5042, Australia

## Abstract

Intraarticular corticosteroid injection (ICSI) is a widely practiced management for hip and knee osteoarthritis. Imposed delays to arthroplasty during coronavirus disease 2019 pandemic have led us to postulate that many patients have opted for recent ICSI. We compared the odds of prosthetic joint infection (PJI) in patients who were or were not administered ICSI within 12 months prior to hip or knee arthroplasty. A systematic search of PubMed, Embase, The Cochrane Library and Web of Science was performed in February 2021, with studies assessing the effect of ICS on PJI rates identified. All studies, which included patients that received ICSI in the 12 months prior to primary hip and knee arthroplasty, were included. In total 12 studies were included: four studies with 209 353 hips and eight studies with 438 440 knees. ICSI administered in the 12 months prior to hip arthroplasty increased the odds of PJI [odds ratio (OR) = 1.17, *P* = 0.04]. This was not the case for knees. Subgroup analysis showed significantly higher odds of PJI in both hip [OR = 1.45, *P* = 0.002] and knee arthroplasty [OR = 2.04; *P* = 0.04] when ICSI was within the preceding 3 months of surgery. A significantly higher odds of PJI were seen in patients receiving ICSI within the 12 months prior to hip arthroplasty. Subgroup analysis showed increased odds of PJI in both hip and knee arthroplasty, in patients receiving ICSI within 3 months prior to their arthroplasty. We recommend delaying knee arthroplasty for at least 3 months after ICSI and possibly longer for hip arthroplasty.

**Level of Evidence:**

Level III - Systematic Review of Level II and III Studies.

## INTRODUCTION

Prosthetic joint infection (PJI) affects between 0.76% and 1.24% of primary hips and knees in the Western world [1]. When PJI occurs, it has potentially devastating consequences, with associated morbidity and a 1-year mortality rate as high as 11% when arthroplasty is revised for PJI [2]. Treatment for PJI varies depending upon the patient profile and the severity of infection, which ranges from implant salvage procedures, such as liner exchange, to single or two-stage revision arthroplasties.

In recent decades, the demand for joint arthroplasty has increased and is projected to continue to rise further [3]. Recent studies project 3.5 million knee and 600 000 total hip arthroplasties will be performed in the USA by 2030 [4]. Accounting for the current incidence of PJI, we expect a disease burden of ∼50 000 PJIs annually. For all patients, arthroplasty is the best considered when non-operative measures have been exhausted. A recommended mode of symptom management in osteoarthritis is via intraarticular corticosteroid injections (ICSIs), often, as part of a multimodal pain management effort [5–9], aiming to achieve symptom control by immunosuppression [10]. Injection into a native joint carries the inherent risk of intraarticular inoculation of pathogens.

It may be theorized that ICSI performed in the months leading up to arthroplasty may risk PJI although the precise time window for this remains unclear. Previous published systematic reviews and meta-analyses on this topic have conflicting results. Two of these meta-analyses concluded an increased risk of subsequent PJI following ICSI [11, 12], while five other studies refuted this conclusion [13–17]. These studies have been criticized for their poor-quality study inclusion and high heterogeneity of their included studies. In this study, we provide an up-to-date systematic review and meta-analysis pertaining to the safe time interval between ICSI and arthroplasty. This is the first systematic review to investigate the temporal relationship between ICSI and odds of acquiring a PJI. Imposed delays to arthroplasty during coronavirus disease (COVID-19) pandemic and with many deferred patients reporting significant symptom progression [18], we postulate that many may have opted for recent ICSI. After pandemic elective arthroplasty restrictions are lifted, most patients will be eager to undergo arthroplasty at the earliest opportunity [19]. This may potentially place them at a higher risk of PJI if our premise is correct.

## MATERIALS AND METHODS

The systematic review was performed based on the recommendations of Preferred Reporting Items for Systematic Reviews and Meta-Analyses (PRISMA).

### Search strategy

We performed a systematic search of the literature across PubMed, Embase, The Cochrane Library and Web of Science from the date of inception of each database through to February 2021. No ‘grey literature’ search was performed. The population of interest was patients with primary hip or knee arthroplasty. Comparisons were made between patients who received ipsilateral ICSI to their native joint within the preceding 12 months of arthroplasty and those who did not. The outcome of interest was the diagnosis of PJI.

A literature search was performed using the following search terms and Boolean operators (‘arthroplasty’ OR ‘joint replacement’) AND (‘injection’ OR ‘steroid’ OR ‘corticosteroid’) AND (‘prosthetic joint infection’ OR ‘periprosthetic infection’ OR ‘infection’). The title and abstracts were then screened by two reviewers independently (T.C. and M.J.) for relevance and consideration into a provisional list. The provisional list was then assessed independently by the two reviewers after reading full text for their potential inclusion. The two reviewers had a consensus on the included articles.

All articles comparing the two comparators on the patient population of interest were included. Studies that included patients who received ipsilateral ICSI more than 12 months prior to arthroplasty with sufficient data in their analysis for us to isolate the patient population of interest were included. Papers that specifically looked into perioperative steroid injection as part of multimodal postoperative analgesia technique, hyaluronic acid injections only, conference abstracts that with insufficient data for extraction, and studies of revision hip or knee arthroplasty patients were excluded. There were no restrictions on included publications, whether based on date of publication, language, study quality or geography.

### Data collection and assessment of risk of bias

Data extraction was performed by the first reviewer (T.C.) and validated by the second reviewer (M.J.). The individual study characteristics and outcomes of interest were assessed. Studies were grouped and assessed separately whether hip or knee arthroplasty. The methodological quality of studies included was assessed independently by both reviewers using the National Institute of Health Quality Assessment Tool for Observational Cohort and Cross-Sectional Studies. Publication bias was assessed using funnel plots.

### Outcomes and statistical analysis

The outcome of interest was the number of PJI in each group. Further subgroup analysis was performed, where study data allowed, regarding the incidence of PJI where ICSI was in the preceding 3 months of arthroplasty. Subgroup analysis was compared against the wider cohort if no readily matched cohort was available. Continuous variables were expressed as mean ± standard error of the mean. Where values were not readily available, this was calculated from the data provided. Statistical analysis was performed using the Mantel–Haenszel method, utilizing either a fixed effect model if the heterogeneity is <50% or a random-effects model if the heterogeneity is >50%. Odds ratio (OR) was used to illustrate the effects of each treatment arm on Forest plots. The corresponding 95% confidence interval (95% CI) and heterogeneity of data (I^2^) are also illustrated in both Forest plots and full text. I^2^ is a scale from 0 to 100% where higher values are associated with greater heterogeneity [20]. The statistical software used in this study was RevMan 5.4 (The Cochrane Collaboration, 2020).

## RESULTS

### Search results

**Fig. 1.**
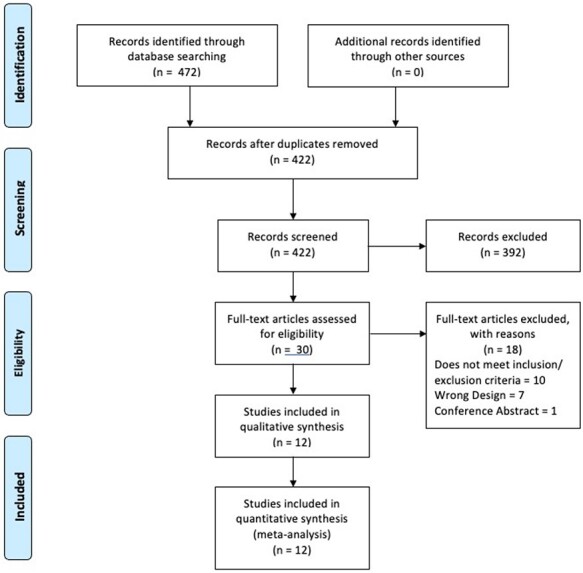
PRISMA flow diagram.

Four hundred and seventy-two studies were identified with the initial search strategy. Fifty duplicate studies were removed. From the remainder, a further 392 studies were excluded by screening titles and abstracts. Twelve studies were included in the final analysis, of which four described PJI in hip arthroplasty [21–24], and the remaining eight described PJI in knee arthroplasty, [25–32] each following intra-articular injections into native joints. The results of our literature search are displayed in Fig. 1.

### Total hip arthroplasty

**Fig. 2.**
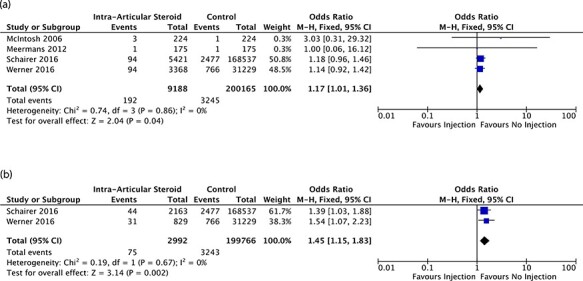
(a) Overall odds of subsequent prosthetic hip joint (hip arthroplasty) infection in patients receiving intra-articular steroid injection to ipsilateral native joint within 12 months prior to replacement. (b) Overall odds of subsequent prosthetic hip joint (hip arthroplasty) infection in patients receiving intra-articular steroid injection to ipsilateral native joint within 3 months prior to replacement.

Four studies with a cumulative sample size of 209 353 hips described the odds of PJI in patients receiving ICSI within 12 months prior to ipsilateral hip arthroplasty [21–24], of which 9188 were in the ICSI group and 200 165 were in the control group. The baseline characteristics of the studies are outlined in Table I. There was no significant heterogeneity between the studies [I^2^ = 0%, *P*-value = 0.86], and hence, a fixed-effect model was used. There was a significant difference in rates of PJI between the two arms [OR = 1.17, 95% CI = 1.01–1.36, *P*-value = 0.04]. The Forest plots for this are shown in Fig. 2a. 

**Table I.** Baseline characteristics of hip arthroplasty studies

		*Cases*					*Controls*		
*Study*	*Study Design (LOE)*	*Number of Hips*	*Age*	*Injection Given*	*Mean Delay*	*Definition of PJI*	*Number of Hips*	*Age*	*Follow-up Period*
McIntosh (2006)	Retrospective matched cohort (level III)	224	70 ± 9.8 years	Type and amount of steroid given was left at the discretion of provider.	3.68 ± 2.66 months	Not stated	224	69 ± 9.6 years	24–60 months
Meermans (2012)	Retrospective matched cohort (level III)	175	66.4 years	80 mg of methylprednisolone and between 1 and 3 mL levobupivacaine	Not stated	Sinus tract communicating with implant, OR Identical pathogen isolated from two or more tissue samples, OR Presence of purulence in joint	175	66.6 years	12–131 months
Schairer (2016)	Retrospective cohort (level III)	5421	66.9	Not stated	Not stated	Hospital readmission with a procedure for infection (irrigation and debridement, implant removal with placement of a cement spacer, or revision hip arthroplasty with a concurrent diagnosis of infection)	168 537	66.6	Up to 12 months
Werner (2016)	Retrospective cohort (level III)	3368	Not stated	Not stated	Not stated	Diagnosis of or procedure for either wound or deep infection 3 or 6 months after THA	31 229	Not stated	Up to 6 months

Two studies [23, 24] with 202 758 hips, of which 2992 were in the ICSI group and 199 766 were in the control group, analyzed the effect of ICSI on the odds of PJI within the 3 months prior to ipsilateral hip arthroplasty. There was no heterogeneity between the studies [I^2^ = 0%, *P*-value = 0.67], and hence, a fixed-effect model was used. There was a significantly increased rate of PJI in patients who received an ICSI in 3 months preceding hip arthroplasty [OR = 1.45, 95% CI = 1.15–1.83, *P*-value = 0.002]. The results of the Forest plots are shown in Fig. 2b.

### Total knee arthroplasty

**Fig. 3.**
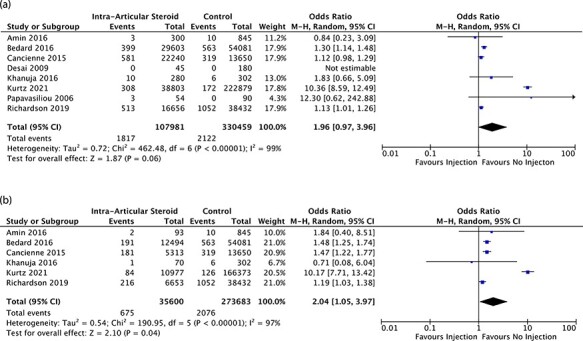
(a) Overall odds of prosthetic knee joint (knee arthroplasty) infection in patients receiving intra-articular steroid injection to ipsilateral native joint within 12 months prior to replacement. (b) Overall odds of prosthetic knee joint (knee arthroplasty) infection in patients receiving intra-articular steroid injection to ipsilateral native joint within 3 months prior to replacement.

Eight studies that enrolled a total of 438 440 cases described the rates of PJI of knee arthroplasty patients who received ICSI within 12 months prior to knee arthroplasty [25–32], of which 107 981 were in the ICSI group and 330 459 were in the control group. The baseline characteristics of these studies are outlined in Table II. There was significant heterogeneity [I^2^ = 99%, *P*-value < 0.00001] between studies, and hence, a random effect model was used. There was no significant difference in rates of PJI between those patients who had and those who had not received ICSI within 12 months prior to their knee arthroplasty [OR = 1.96, 95% CI = 0.97–3.96, *P*-value = 0.06]. The results of the Forest plots are shown in Fig. 3a.

**Table II.** Baseline characteristics of knee arthroplasty series

		*Cases*					*Controls*		
*Study*	*Study Design (LOE)*	*Number of Knees*	*Age*	*Injection Given*	*Mean Delay*	*Definition of PJI*	*Number of Knees*	*Age*	*Follow-up Period*
Amin (2016)	Retrospective cohort (level III)	300	Not stated	Not stated	Not stated	MSIS criteria as assessed by two senior surgeons	845	64.14 years	Not stated
Bedard (2016)	Retrospective matched cohort (level III)	29 603	Not stated	Not stated	Not stated	Patients identified to have undergone operative management related to Total Knee Arthroplasty (TKA) surgical site infection	54 081	Not stated	6 months
Cancienne (2015)	Retrospective matched cohort (level III)	22 240	Not stated	Not stated	Not stated	Diagnosis or procedure for wound or deep infection within 3 or 6 months after Total Knee Arthroplasty (TKA)	13 650	Not stated	6 months
Desai (2009)	Prospective matched cohort (level II)	45	Not stated	Depomedrone 40 mg + Chirocaine	Not stated	Cases with positive swab cultures or tissue biopsy from deep tissues and underwent washout/ debridement as a result, OR patients who underwent revision surgery for infection	180	72 years	12–72 months
Khanuja (2016)	Prospective cohort (level II)	280	Not stated	Triamcinolone acetonide 1 mL + Xylocaine (4%)	Not stated	Sinus tract to prosthesis Pathogen isolated by culture from two or more deep samples Four of the following: raised ESR or CRP; raised synovial leukocyte count; raised synovial neutrophil percentage; purulence from joint; pathogen isolated from single deep specimen; more than 5 neutrophils per HPF	302	65	21–66 months
Kurtz (2021)	Retrospective cohort (level III)	38 803	Not stated	Either corticosteroid injection or corticosteroid mixed with hyaluronic acid	Not stated	Patients identified using diagnostic code for infection as well as concurrent procedural code for either revision arthroplasty, arthrotomy, or spacer insertion	222 879	Not stated	24 months
Papavasiliou (2006)	Retrospective cohort (level III)	54	Not stated	Not stated	Not stated	Purulent drainage from depths of incision Positive culture from aseptically aspirated fluid or deep tissue biopsy, or pus cells on microscopy Deep incision that has spontaneously dehisced or opened by a surgeon when the patient was febrile An abscess or evidence of infection involving deep tissues seen during reoperation Diagnosis by attending clinician	90	Not stated	Not stated
Richardson (2019)	Retrospective cohort (level III)	16 656	Not stated	Not stated	Not stated	Diagnosis or procedure for wound or deep infection within 6 months after Total Knee Arthroplasty (TKA)	38 432	Not stated	6 months

Six of those eight studies with a total of 309 283 knees, of which 35 600 were in the ICSI group and 273 683 were in the control group, described the rates of PJI in patients who received ICSI within 3 months prior to arthroplasty [25–27, 29, 30, 32]. There was significant heterogeneity [I^2^ = 97%, *P*-value < 0.00001] between studies, and hence, a random effect model was used. There was a significantly increased odds of PJI in those who had received ICSI within 3 months prior to arthroplasty, compared to those who had not [OR = 2.04, 95% CI = 1.05–3.97, *P*-value = 0.04]. The Forest plots are shown in Fig. 3b.

### Risk of bias analysis

Risk of bias in individual studies was analyzed using the National Institute of Health Quality Assessment Tool for Observational Cohort and Cross-Sectional Studies. The results are displayed in Table III. No studies provided any justification for sample size or the number of ICSI. Concerningly, a study by Amin *et al*. categorized patients into one of three groups: no injection, steroid injection or viscosupplementation injection. However, the group classification was based upon which injection the patient last had, which may have led to significant bias in the results [25]. It was unclear from all studies whether outcome assessors were blinded to the exposure status of the participants.

**Table III.** Risk of bias analysis using the National Institute of Health assessment tool for observational cohort and cross-sectional studies

	*McIntosh*	*Meermans*	*Schairer*	*Werner*	*Amin*	*Bedard*	*Cancienne*	*Desai*	*Khanuja*	*Kurtz*	*Papavasiliou*	*Richardson*
	*(2006)*	*(2012)*	*(2016)*	*(2016)*	*(2016)*	*(2016)*	*(2015)*	*(2009)*	*(2016)*	*(2021)*	*(2006)*	*(2019)*
Was the research question or objective in this paper clearly stated?	Y	Y	Y	Y	Y	Y	Y	Y	Y	Y	Y	Y
Was the study population clearly specified and defined?	Y	Y	Y	Y	Y	Y	Y	Y	Y	Y	Y	Y
Was the participation rate of eligible persons at least 50%?	Y	Y	Y	Y	Y	Y	Y	Y	Y	Y	Y	Y
Were all the subjects selected or recruited from the same or similar populations (including the same time period)? Were inclusion and exclusion criteria for being in the study prespecified and applied uniformly to all participants?	Y	Y	Y	Y	Y	Y	Y	Y	Y	Y	Y	Y
Was a sample size justification, power description, or variance and effect estimates provided?	N	N	N	N	N	N	N	N	N	N	N	N
For the analyses in this paper, were the exposure(s) of interest measured prior to the outcome(s) being measured?	N	N	N	N	N	N	N	Y	Y	N	N	N
Was the timeframe sufficient so that one could reasonably expect to see an association between exposure and outcome if it existed?	Y	Y	Y	Y	Y	Y	Y	Y	Y	Y	Y	Y
For exposures that can vary in amount or level, did the study examine different levels of the exposure as related to the outcome (e.g. categories of exposure, or exposure measured as continuous variable)?	N	N	N	N	N	N	N	N	N	N	N	N
Were the exposure measures (independent variables) clearly defined, valid, reliable and implemented consistently across all study participants?	Y	Y	Y	Y	Y	Y	Y	Y	Y	Y	Y	Y
Was the exposure(s) assessed more than once over time?	N	N	N	N	N	N	N	N	N	N	N	N
Were the outcome measures (dependent variables) clearly defined, valid, reliable, and implemented consistently across all study participants?	Y	Y	Y	Y	Y	Y	Y	Y	Y	Y	Y	Y
Were the outcome assessors blinded to the exposure status of participants?	NS	NS	NS	NS	NS	NS	NS	NS	NS	NS	NS	NS
Was loss to follow-up after baseline 20% or less?	Y	Y	Y	Y	Y	Y	Y	Y	Y	Y	Y	Y
Were key potential confounding variables measured and adjusted statistically for their impact on the relationship between exposure(s) and outcome(s)?	Y	Y	Y	Y	N	Y	Y	Y	Y	Y	Y	Y

**Fig. 4.**
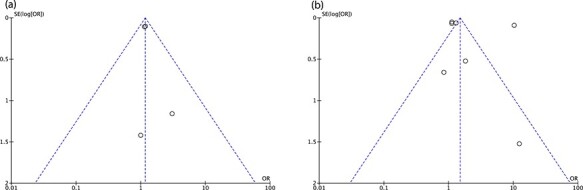
(a) Funnel plots for hip arthroplasty studies. (b) Funnel plots for knee arthroplasty studies.

Funnel plots were constructed to assess the risk of publication bias. There was symmetry in the hip arthroplasty studies (Fig. 4a), suggesting a low risk of publication bias. Knee arthroplasty studies on the other hand demonstrated asymmetry, suggesting significant publication bias (Fig. 4b).

## DISCUSSION

The results of our systematic review and meta-analysis showed that there were increased odds of PJI when ICSI was administered within 12 months prior to hip arthroplasty. In knee arthroplasty, the odds of PJI were increased, although not significantly, when ICSI was administered 12 months prior to arthroplasty and should be interpreted with caution given high heterogeneity. Further subgroup analysis revealed significantly increased odds of PJI for both hip and knee arthroplasty when ICSI was administered within 3 months prior to arthroplasty. In hip arthroplasty, there appeared to be a temporal relationship, whereby the odds of PJI decreased from 1.45 at 3 months prior to 1.17 within 12 months prior to their arthroplasty. We postulate our significant findings to be due to the immune-modulatory effects of steroids or the inherent risk of inadvertent inoculation of pathogens at ICSI [32–35]. Both these possible scenarios warrant wider investigation. Furthermore, we believe a broader investigation may be warranted into the benefit, risk and complication of intraarticular platelet-rich plasma (PRP) or bone marrow aspirate concentrate (BMAC) injection to the arthritic hip and knee joint, a rapidly embraced clinic treatment in this particular patient population [36, 37]. Although there is weak evidence for the use of BMAC and PRP in hip or knee osteoarthritis [38, 39], the infection rates of each of these treatment modalities are yet to be established.

This study is even more relevant at present, with critical demands placed upon overstretched healthcare systems across the world amidst the current COVID-19 pandemic; there has been significant disruption to elective arthroplasty schedules around the world. To appreciate the magnitude of this backlog, an estimated 30 000 primary and 3000 revision hip and knee arthroplasty procedures were cancelled in each week of imposed elective surgery restrictions within the United States alone [40]. During the early stages of the pandemic, there was an initial widespread abandonment of ICSI; however, as the pandemic progressed, there have been peak body recommendations and multiple other studies advocating the safe utility of ICSI in hip and knee osteoarthritis [18, 41–44]. Brown *et al*. surveyed over 800 patients across 15 institutions in the USA, each planned for elective hip or knee arthroplasty but rescheduled due to the pandemic. They found that 54% of respondents reported worsening arthritis symptoms during this period and 87% respondents remained eager to have their arthroplasty soonest deemed safe [19]. In this setting, it is highly likely that such patients with daily intrusive pain of arthritis may opt to temporize symptoms with ICSI [42]. This may risk patients having ICSI in narrow and unsafe timeframes in relation to their rescheduled arthroplasty. Clinicians and patients should be educated and alerted to this potential risk and be vigilant when rescheduling long-awaited arthroplasty.

Varied case definition for PJI between studies is seen in Tables I and 2. Some of the large studies utilized coding systems, either state-wide [23, 30] or insurance based [24, 26, 32], to identify patients with PJI. These systems, although efficient, are prone to potential erroneous under-reporting of cases of PJI. Other studies analyzed cases on individual merit and utilized variations of the Musculoskeletal Infection Society (MSIS) criteria. Our study has inherent limitations by virtue of design and inclusion criteria. Our study cohorts may have included those with undeclared confounding interventions with potential to influence PJI. These modalities may have included intraarticular synovial fluid supplementation [45], intraarticular injectable cellular therapies (such as PRP or BMAC) [46] and potentially acupuncture [47]. Additionally, the number of ICSI provided in the study period was unclear in most studies. Additionally, variation of follow-up intervals (ranging from 6 to 131 months) in included studies may have affected their reported rates of PJI.

Only two of the 12 studies included in our final analysis were prospective cohort studies. The remaining studies were either retrospective cohort studies or retrospective matched cohort studies, which provided Level III evidence. There were no randomized controlled trials that were identified in this systematic review. There was also potentially significant publication bias in the knee arthroplasty studies, as evidenced by the funnel plot analysis in Fig. 4b, despite our attempt to limit publication bias by including wider databases, such as Web of Science. Other causes for funnel plot asymmetry, such as selective outcome reporting and selective analysis reporting may also be present [48]. Future systematic reviews and meta-analysis of this topic should consider obtaining unpublished data to help reduce publication bias. Lastly, there may be the introduction of bias in our subgroup analyses, as our comparator group was not a matched cohort in all studies apart from Kurtz *et al*. [30]. A singular definition of PJI and higher-quality prospective studies should provide more robust data to enhance future best practice.

In conclusion, within the confines of our study, ICSI performed within 3 months prior to arthroplasty significantly increased the odds of PJI in both hip [OR = 1.45, *P*-value = 0.002] and knee arthroplasty [OR = 2.04; *P*-value = 0.04] and, thus, best avoided. There was a significant increase in odds of PJI when ICSI was administered 12 months prior to hip arthroplasty [OR = 1.17, *P*-value = 0.04]. Patients considering hip arthroplasty within 12 months of ICSI should be appropriately counseled based on this finding. This is of particular importance in the months ahead, if we are to collectively and safely guide the prompt delivery of arthroplasty once COVID-19 pandemic arthroplasty restrictions are lifted. Higher-quality prospective studies with a standardized definition of PJI may reliably enhance our future understanding of the implications and safety of ICSI.

## Data Availability

Not Applicable.

## References

[1] Springer BD, Cahue S, Etkin CD et al. Infection burden in total hip and knee arthroplasties: an international registry-based perspective. *Arthroplasty Today* 2017; 3: 137–40. 28695187 10.1016/j.artd.2017.05.003 PMC5485227

[2] Gundtoft PH, Pedersen AB, Varnum C et al. Increased mortality after prosthetic joint infection in primary THA. *Clin Orthop Relat Res* 2017; 475: 2623–31. 28236084 10.1007/s11999-017-5289-6 PMC5638726

[3] Ackerman IN, Bohensky MA, Zomer E et al. The projected burden of primary total knee and hip replacement for osteoarthritis in Australia to the year 2030. *BMC Musculoskelet Disord* 2019; 20: 90. 10.1186/s12891-019-2411-9 PMC6387488 30797228

[4] Masaracchio M, Hanney WJ, Liu X et al. Timing of rehabilitation on length of stay and cost in patients with hip or knee joint arthroplasty: a systematic review with meta-analysis. *PLoS One* 2017; 12: e0178295. 10.1371/journal.pone.0178295 PMC5456061 28575058

[5] Creamer P . Intra-articular corticosteroid treatment in osteoarthritis. *Curr Opin Rheumatol* 1999; 11: 417–21. 10503664 10.1097/00002281-199909000-00016

[6] Chao J, Wu C, Sun B et al. Inflammatory characteristics on ultrasound predict poorer longterm response to intraarticular corticosteroid injections in Knee Osteoarthritis. *J Rheumatol* 2010; 37: 650–5. 20080918 10.3899/jrheum.090575

[7] Chandrasekaran S, Lodhia P, Suarez-Ahedo C et al. Symposium: evidence for the use of intra-articular cortisone or hyaluronic acid injection in the hip. *J Hip Preserv Surg* 2016; 3: 5–15. 27026814 10.1093/jhps/hnv020 PMC4808252

[8] Tangtiphaiboontana J, Zhang AL, Pandya NK. Outcomes of intra-articular corticosteroid injections for adolescents with hip pain. *J Hip Preserv Surg* 2018; 5: 54–9. 29423251 10.1093/jhps/hnx027 PMC5798032

[9] Zhang AL, Tangtiphaiboontana J, Pandya N. Outcomes of intra-articular corticosteroid injections for adolescents with hip pain. *J Hip Preserv Surg* 2016; 3.doi: 10.1093/jhps/hnw030.018. PMC5798032 29423251

[10] Ayhan E, Kesmezacar H, Akgun I. Intraarticular injections (corticosteroid, hyaluronic acid, platelet rich plasma) for the knee osteoarthritis. *World J Orthop* 2014; 5: 351–61. 25035839 10.5312/wjo.v5.i3.351 PMC4095029

[11] Xing D, Yang Y, Ma XL et al. Dose intraarticular steroid injection increase the rate of infection in subsequent arthroplasty: grading the evidence through a meta-analysis. *J Orthop Surg Res* 2014; 9: 107.doi: 10.1186/s13018-014-0107-2. PMC4245809 25391629

[12] Meng FT, Gong BB, Yang G et al. Intra-articular steroid injections and risk of infection following total hip replacement or total knee replacement: a meta-analysis of cohort studies. *Int J Clin Exp Med* 2016; 9: 11002–8.

[13] Ellerby N, Hider S, Mallen C et al. Does intra-articular corticosteroid injection in the pre-operative period increase the risk of joint infection following hip or knee arthroplasty? A systematic review and meta-analysis. *Rheumatology (United Kingdom)* 2014; 53: i132.

[14] Wang QQ, Jiang X, Tian W. Does previous intra-articular steroid injection increase the risk of joint infection following total hip arthro plasty or total knee arthroplasty? A meta-analysis. *Med Sci Monitor* 2014; 20: 1878–83. 10.12659/MSM.890750 PMC4206397 25298367

[15] Charalambous CP, Prodromidis AD, Kwaees TA. Do intra-articular steroid injections increase infection rates in subsequent arthroplasty? A systematic review and meta-analysis of comparative studies. *J Arthroplasty* 2014; 29: 2175–80. 25201257 10.1016/j.arth.2014.07.013

[16] Pereira LC, Kerr J, Jolles BM. Intra-articular steroid injection for osteoarthritis of the hip prior to total hip arthroplasty is it safe? A systematic review. *Bone Joint J* 2016; 98B: 1027–35. 10.1302/0301-620X.98B8.37420 27482013

[17] McMahon SE, LeRoux JA, Smith TO et al. Total joint arthroplasty following intra-articular steroid injection: a literature review. *Acta Orthop Belg* 2013; 79: 672–9. 24563973

[18] Cisternas AF, Ramachandran R, Yaksh TL et al. Unintended consequences of COVID-19 safety measures on patients with chronic knee pain forced to defer joint replacement surgery. *Pain Rep* 2020; 5: e855. 10.1097/PR9.0000000000000855 PMC7553566 33134751

[19] Brown TS, Bedard NA, Rojas EO et al. The effect of the COVID-19 pandemic on hip and knee arthroplasty patients in the United States: a multicenter update to the previous survey. *Arthroplasty Today* 2021. 10.1016/j.artd.2020.11.025 10.1016/j.artd.2020.11.025 PMC7713541 33294537

[20] Higgins JPT, Thompson SG. Quantifying heterogeneity in a meta‐analysis. *Stat Med* 2002; 21: 1539–58. 12111919 10.1002/sim.1186

[21] McIntosh AL, Hanssen AD, Wenger DE et al. Recent intraarticular steroid injection may increase infection rates in primary THA. *Clin Orthop Relat Res* 2006; 451: 50–4.doi: 10.1097/01.blo.0000229318.51254.79. 16906098

[22] Meermans G, Corten K, Simon JP. Is the infection rate in primary THA increased after steroid injection? *Clin Orthop Relat Res* 2012; 470: 3213–9. 22610526 10.1007/s11999-012-2390-8 PMC3462846

[23] Schairer WW, Nwachukwu BU, Mayman DJ et al. Preoperative hip injections increase the rate of periprosthetic infection after total hip arthroplasty. *J Arthroplasty* 2016; 31: 166–9e1. 27221820 10.1016/j.arth.2016.04.008

[24] Werner BC, Cancienne JM, Browne JA. The timing of total hip arthroplasty after intraarticular hip injection affects postoperative infection risk. *J Arthroplasty* 2016; 31: 820–3. 26803711 10.1016/j.arth.2015.08.032

[25] Amin NH, Omiyi D, Kuczynski B et al. The risk of a deep infection associated with intraarticular injections before a total knee arthroplasty. *J Arthroplasty* 2016; 31: 240–4. 26432675 10.1016/j.arth.2015.08.001

[26] Bedard NA, Pugely AJ, Elkins JM et al. The John N. Insall award do intraarticular injections increase the risk of infection after TKA? *Clin Orthop Relat Res* 2017; 475: 45–52. 26970991 10.1007/s11999-016-4757-8 PMC5174022

[27] Cancienne JM, Werner BC, Luetkemeyer LM et al. Does timing of previous intra-articular steroid injection affect the post-operative rate of infection in total knee arthroplasty? *J Arthroplasty* 2015; 30: 1879–82. 26071248 10.1016/j.arth.2015.05.027

[28] Desai A, Ramankutty S, Board T et al. Does intraarticular steroid infiltration increase the rate of infection in subsequent total knee replacements? *Knee* 2009; 16: 262–4. 19138855 10.1016/j.knee.2008.12.002

[29] Khanuja HS, Banerjee S, Sodhi GS et al. Do prior intra-articular corticosteroid injections or time of administration increase the risks of subsequent periprosthetic joint infections after total knee arthroplasty? *J Long Term Eff Med Implants* 2016; 26: 191–7. 28134600 10.1615/JLongTermEffMedImplants.2016014045

[30] Kurtz SM, Mont MA, Chen AF et al. Intra-articular corticosteroid or hyaluronic acid injections are not associated with periprosthetic joint infection risk following total knee arthroplasty. *J Knee Surg* 2021. 10.1055/s-0040-1721128 10.1055/s-0040-1721128 33389729

[31] Papavasiliou AV . Infection in knee replacements after previous injection of intra-articular steroid - reply. *J Bone Joint Surg* 2007; 89B: 422. 10.1302/0301-620X.89B3.19287 17356167

[32] Richardson SS, Schairer WW, Sculco TP et al. Comparison of infection risk with corticosteroid or hyaluronic acid injection prior to total knee arthroplasty. *J Bone Joint Surg Am* 2019; 101: 112–8. 30653040 10.2106/JBJS.18.00454

[33] Habib GS . Systemic effects of intra-articular corticosteroids. *Clin Rheumatol* 2009; 28: 749–56. 19252817 10.1007/s10067-009-1135-x

[34] Wang Y, Leng V, Patel V et al. Injections through skin colonized with Staphylococcus aureus biofilm introduce contamination despite standard antimicrobial preparation procedures. *Sci Rep* 2017; 7: 45070. 10.1038/srep45070 PMC5362901 28332593

[35] Xu C, Peng H, Chai W et al. Inadvertent introduction of tissue coring into joints during arthrocentesis: an experimental study. *Med Sci Monit* 2017; 23: 3571–7. 28733573 10.12659/MSM.905590 PMC5536130

[36] Migliorini F, Driessen A, Quack V et al. Comparison between intra-articular infiltrations of placebo, steroids, hyaluronic and PRP for knee osteoarthritis: a Bayesian network meta-analysis. *Arch Orthop Trauma Surg* 2020. 10.1007/s00402-020-03551-y 10.1007/s00402-020-03551-y 32725315

[37] Chahla J, Dean CS, Moatshe G et al. Concentrated bone marrow aspirate for the treatment of chondral injuries and osteoarthritis of the knee: a systematic review of outcomes. *Orthop J Sports Med* 2016; 4: 2325967115625481. 10.1177/2325967115625481 PMC4714134 26798765

[38] Sullivan SW, Aladesuru OM, Ranawat AS et al. The use of biologics to improve patient-reported outcomes in hip preservation. *J Hip Preserv Surg* 2021; hnab028 .doi: 10.1093/jhps/hnab028. PMC8460156 34567595

[39] Delanois RE, Etcheson JI, Sodhi N et al. Biologic therapies for the treatment of knee osteoarthritis. *J Arthroplasty* 2019; 34: 801–13. 30612835 10.1016/j.arth.2018.12.001

[40] Chen AZ, Shen TS, Bovonratwet P et al. Total joint arthroplasty during the COVID-19 pandemic: a scoping review with implications for future practice. *Arthroplasty Today* 2021; 8: 15–23. 33521188 10.1016/j.artd.2020.12.028 PMC7836630

[41] Morgan C, Dattani R. Should I use steroid injections to treat shoulder pain during the COVID-19 pandemic? *JSES Int* 2020; 4: 709–12. 32924020 10.1016/j.jseint.2020.07.023 PMC7480274

[42] McKean D, Chung SL, Fairhead R et al. Corticosteroid injections during the COVID-19 pandemic. *Bone and Joint Open* 2020; 1: 605–11. 33215158 10.1302/2633-1462.19.BJO-2020-0130.R1 PMC7659632

[43] FPM response to concern related to the safety of steroids injected as part of pain procedures during the current COVID-19 virus pandemic [press release]. United Kingdom: Royal College of Anaethetists. 17 March 2020. https://fpm.ac.uk/sites/fpm/files/documents/2020-03/FPM-COVID-19-Steroid-Statement-2020-v2.pdf.

[44] Corticosteroid use for musculoskeletal and rheumatic conditions during COVID-19 pandemic. United Kingdom: British Orthopaedic Association, Association of Chartered Physiotherapists in Orthopaedic medicine and injection therapy, British Association of Spinal Surgeons and the British Society for Rheumatology. 23 March 2020. https://www.boa.ac.uk/uploads/assets/30a67bae-1e3a-4b76-bf97b7f86600230b/Corticosteroid-use-for-musculoskeletal-and-rheumatic-conditions-during-COVID-19-Pandemic-V1.pdf.

[45] Colen S, Hoorntje A, Maeckelbergh L et al. Intra-articular hyaluronic acid injections less than 6 months before total hip arthroplasty: is it safe? A retrospective cohort study in 565 patients. *J Arthroplasty* 2021; 36: 1003–8. 33097337 10.1016/j.arth.2020.09.024

[46] Piuzzi NS, Emara A, Chahla J et al. Ethical and practical considerations for integrating cellular (“stem cell”) therapy into clinical practice. *Curr Rev Musculoskelet Med* 2020; 13: 525–9. 32468421 10.1007/s12178-020-09647-7 PMC7340700

[47] Nakajima A, Kaneyama R, Watanabe H et al. Acupuncture needle-associated prosthetic knee infection after total knee arthroplasty. *Mod Rheumatol* 2010; 20: 627–31. 20617357 10.1007/s10165-010-0331-5

[48] Sterne JAC, Sutton AJ, Ioannidis JPA et al. Recommendations for examining and interpreting funnel plot asymmetry in meta-analyses of randomised controlled trials. *BMJ* 2011; 343: d4002. 10.1136/bmj.d4002 21784880

