# Corrigendum to: Safety of intraarticular corticosteroid injection preceding hip and knee arthroplasty: a systematic review and meta-analysis amid resolving COVID-19 arthroplasty restrictions

**DOI:** 10.1093/jhps/hnab072

**Published:** 2021-10-04

**Authors:** Tim Cheok, Matthew Jennings, Alessandro Aprato, Narlaka Jayasekera, Ruurd L Jaarsma

*Journal of Hip Preservation Surgery*, https://doi.org/10.1093/jhps/hnaa064

Upon the original publication of this article, there was an error in co-author Ruurd L. Jaarsma’s affiliation. The full affiliation should read: ‘Flinders Medical Centre, Flinders Drive, Bedford Park, South Australia 5042, Australia’ instead of ‘Department of Trauma and Orthopaedics, Alice Springs Hospital, 6, Gap Road, Northern Territory 0870, Australia’ and ‘Department of Trauma and Orthopaedics, Flinders drive Medical Centre, Bedford Park, Adelaide, South Australia 5042, Australia’.

This error has now been corrected.

